# Case report: Intraosseous hemangioma of the lateral clavicle treated by surgical resection and reconstructed by three-dimensional-printed personalized prosthesis

**DOI:** 10.3389/fbioe.2022.1085674

**Published:** 2023-01-09

**Authors:** Zhuangzhuang Li, Chang Zou, Minxun Lu, Yuqi Zhang, Taojun Gong, Jie Wang, Yi Luo, Yong Zhou, Li Min, Chongqi Tu

**Affiliations:** ^1^ Department of Orthopedics, Orthopaedic Research Institute, West China Hospital, Sichuan University, Chengdu, China; ^2^ Model Worker and Craftsman Talent Innovation Workshop of Sichuan province, Chengdu, Sichuan, China

**Keywords:** 3D-printed prosthesis, clavicle, tumor resection, intraosseous hemangioma, bone defect

## Abstract

**Background:** Intraosseous hemangiomas occurring the clavicle is uncommon. Reconstruction of the clavicle is suggested to maintain the normal shoulder joint function and prevent adverse outcomes. Complex anatomy shape of the clavicle remains a great challenge for prosthetic reconstruction of the clavicle.

**Case presentation:** A 37-year-old female with no conclusive history of trauma presented with progressive mass at the right lateral clavicle for 5 years. The patient was treated by surgical resection and reconstructed by three-dimensional-printed personalized prosthesis. Postoperatively radiographic examinations revealed a good position of the prosthesis, neither breakage nor loosening was detected. The right shoulder mobility returned to approximate level of preoperative shoulder 2 months after surgical reconstruction, with the range of motion of flexion 80°, extension 40°, abduction 80°, adduction 30°, external rotation 55°, and internal rotation 60°. The patient maintained the normal shoulder function during the 48 months follow-up period. There was no pain during shoulder motion. The Musculoskeletal Tumor Society Score (MSTS) score was 29 and the Functional Evaluation Form recommended by the American Shoulder and Elbow Surgeons (ASES) score was 95.

**Conclusion:** 3D-printed personalized prosthesis is a good option to reconstruct the lateral clavicle bone defect and restore the shoulder support structure. It maintains the normal shoulder joint function and avoids adverse effects on daily activities after claviculectomy.

## 1 Introduction

Intraosseous hemangiomas are benign tumors composed of neoplastic blood vessels (Rickert and Meurer, 2017), representing approximately 1% of all bone tumors (Gologorsky et al., 2013). The common sites of intraosseous hemangiomas are the vertebral column and skull. In contrast, hemangiomas occurring the clavicle is uncommon and up to our knowledge, there are few cases have been reported (Fráter et al., 1971; Fnini et al., 2011; Matsumoto et al., 2014). With regard to the treatment, surgical resection is recommended for tumor local control. However, the resulting cosmetic and functional impairment of clavicle resection may be significant with sloped shoulder, limited motion of the shoulder joint, and restriction of daily activities (Chen et al., 2021). Therefore, reconstruction of the clavicle is suggested to maintain the normal shoulder joint function and prevent adverse outcomes.

Currently, the major materials of bone defect repairment after clavicle resection include autograft bone (rib, fibula) and allograft bone (Lin et al., 2014). Reconstruction with autograft can achieve good integration because of its ideal osteogenic, osteoinductive, and osteoconductive properties (Rodríguez-Nogué et al., 2020). But this method is associate with morbidities of the donor site, trauma and pain. Furthermore, autograft is difficult to repair the shape of shoulder. Allograft has a chance to restore shoulder contour and shoulder support structure (Momberger et al., 2000; Li et al., 2011). However, the relatively high risk of postoperative complications restricts its clinical application, such as infection, non-union and inferior integration. Due to the advantages of satisfactory mechanical properties and no risk of disease transmission, prosthesis has received considerate attention in reconstructing limb bone defect. Nevertheless, complex anatomy shape of the clavicle remains a great challenge for traditional manufacturing process to fabricate such implantation. Compared to traditional manufacturing process, three-dimensional-(3D)-printing technology simplifies the processing steps and considerably reduces processing time in fabrication such kind of implantation (Fan et al., 2015).

In this case, we present a patient with intraosseous hemangiomas of the lateral clavicle and coracoid process. The mass of lateral clavicle affected the aesthetic and slightly restricted shoulder movement. We treated the patient through surgical resection of the tumors and reconstructed the lateral clavicle using a 3D-printed personalized prosthesis. The prosthesis was designed by mirroring corresponding part of normal clavicle and therefore closer to patient’s anatomy shape. The aims of the present case were to describe the reconstruction process and assess the clinical outcomes of this reconstruction.

## 2 Case presentation

### 2.1 History

**FIGURE 1:**
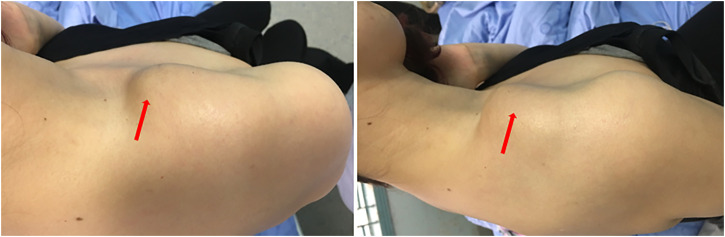
Photograph of the patient showing the right lateral clavicle mass.

A 37-year-old female with no conclusive history of trauma presented with progressive mass at the right lateral clavicle for 5 years. The patient had complained discomfort and pain during movement of the right shoulder for 2 months. And then, the patient visited our institution for treatment. Physical examination indicated a hard mass located lateral clavicle area approximately 8 × 4 × 4 cm in size, with no overlying erythema (Figure 1). The shoulder movement was sightly restricted. No signs of local infection were present. Laboratory values were within the normal ranges.

**FIGURE 2:**
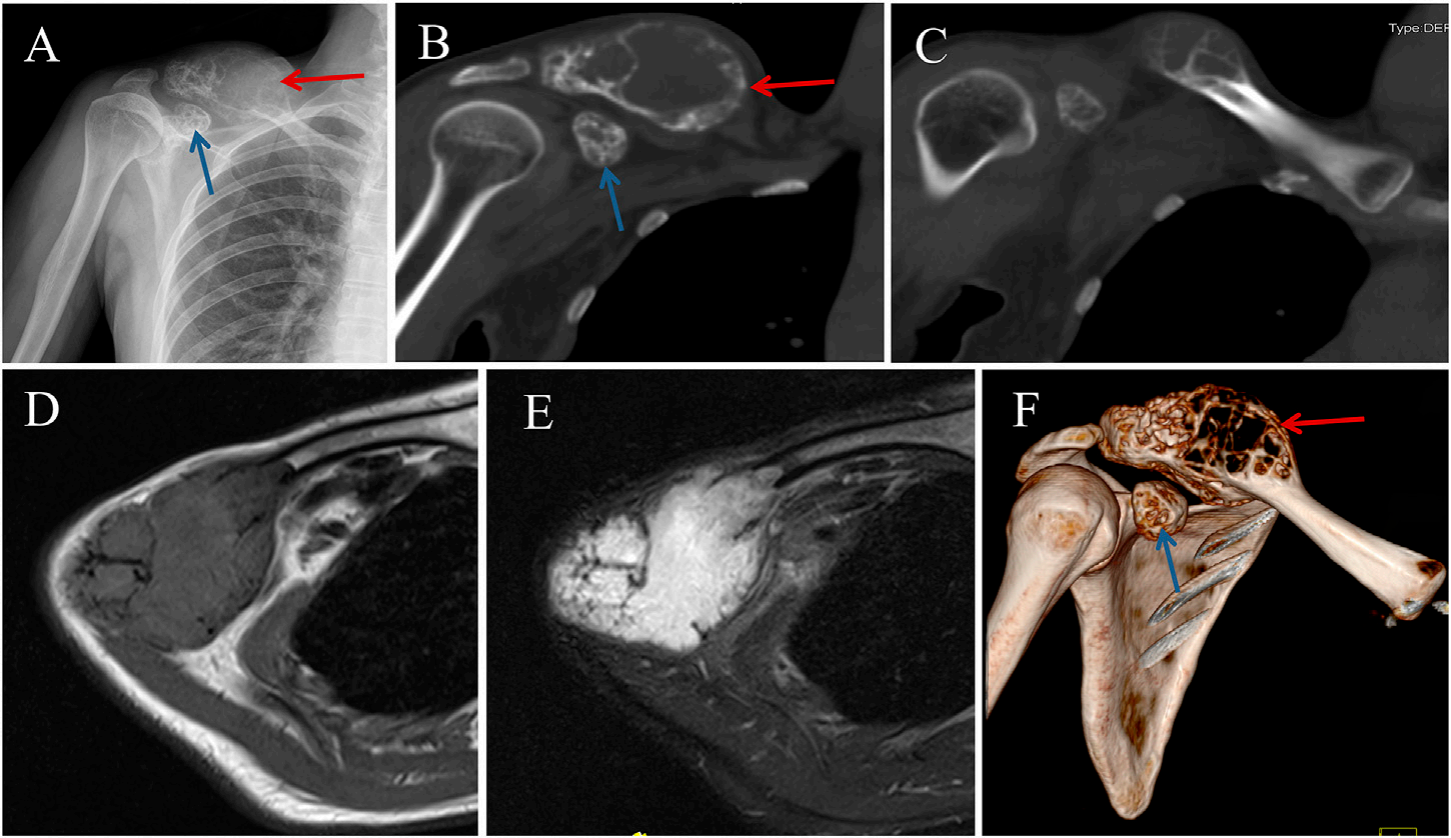
Preoperative image data of the patient. **(A)** Anteroposterior X-ray of the right clavicle showing an large and expansive lesion (Red Arrow), with another lesion involving coracoid process (Blue Arrow). **(B**, **C)** Computed tomography (CT) images showing osteolytic lesions with cortex-destructing. Magnetic resonance imaging showing the expansive lump with isointensity to muscle on a **(D)** T1-weighted image and homogeneous hyperintensity on a T2-weighted image **(E)**. **(F)** 3D model of the shoulder reconstructed from CT scans.

The X-ray and computed tomography (CT) scan revealed an expansive and osteolytic lesion with cortex-destructing in the right lateral clavicle (Figure 2). There was another lesion involving coracoid process, approximately 3 × 2.2 × 1.5 cm in size. The patient subsequent underwent open biopsy, and the result suggested intraosseous hemangioma. After the informed written consent was obtained from patient, surgical resection of the lesions and reconstruction of the lateral clavicle with a 3D-printed personalized prosthesis were planned.

### 2.2 Prosthesis design and fabrication

**FIGURE 3:**
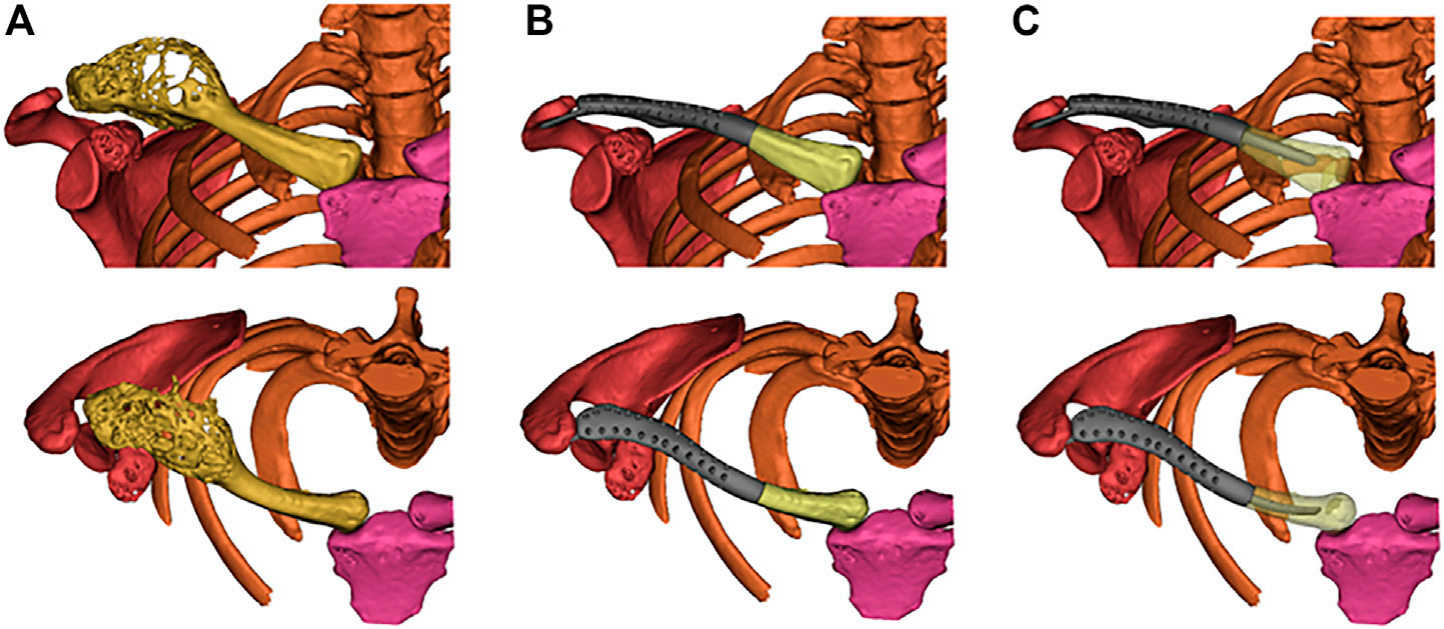
**(A)** Reconstructed virtual 3D tumors and clavicle models. **(B)** The virtual 3D personalized prosthesis model. **(C)** Simulated implantation of the prosthesis with a curved intramedullary stem.

**FIGURE 4:**
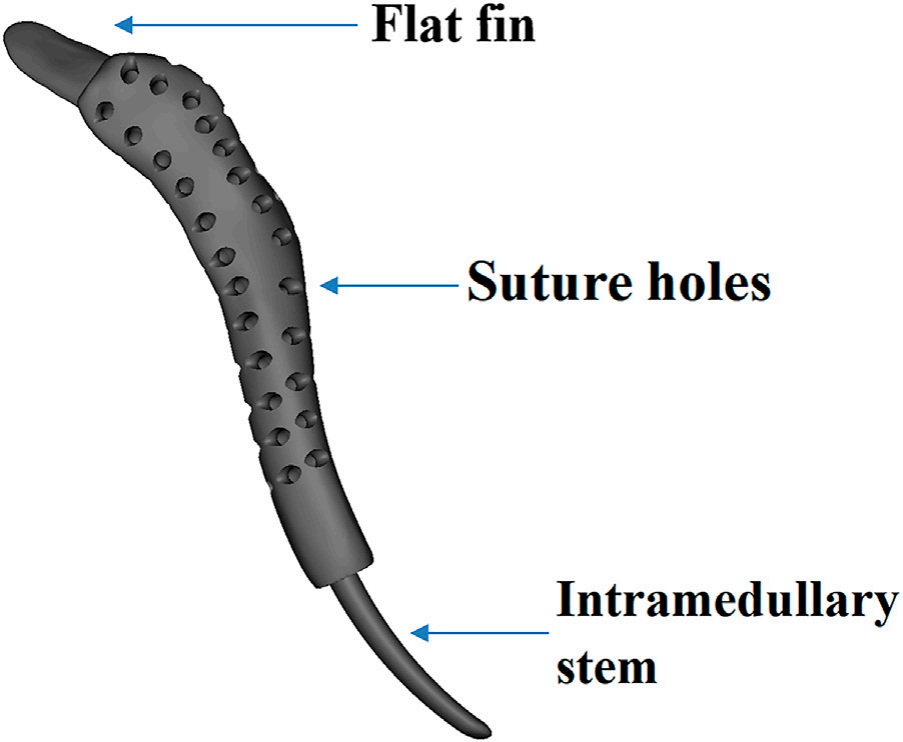
Profile of the prosthesis.

The prosthesis was designed by our clinic team and fabricated by Chunli Co., Ltd. (Tongzhou, Beijing, People’s Republic of China). CT date was obtained in DICOM format and imported into Materialize Mimics V17.0 (Materialize Corp., Leuven, Belgium) software for building virtual 3D tumor and clavicle models. Firstly, segmenting out the bone *via* thresholding, and then the region-of-interest was deleted. Lastly, “Region Grow” was used to segment out the bone (Figure 3). The osteotomy level was set as 10 mm beyond boundary, and the osteotomy was simulated. And then, the models were stored in STL format and imported into Geomagic Studio software (Geomagic Inc., Morrisville, United States) for designing the prosthesis. The preliminary prosthesis shape was designed by mirroring the contralateral normal clavicle model (Figure 4). After that, removing unnecessary features and smoothing the surface of prosthesis were performed. And then, specific features, such as suture holes for soft tissue repairing were set on the prosthetic body. At the medial side of the prosthetic body, an intramedullary stem with a curvature consistent with the medial clavicle was designed for insertion into the residual clavicle. While a flat fin was designed at lateral side of the prosthetic body. Medical-grade Ti-6Al-4V powder was used as the printing raw material. The prosthesis was fabricated using an electron beam melting (EBM) machine (ARCAM Q10plus, Mölndal, Sweden), which is a powder bed fusion technique. The yield strength of the formed titanium alloy was approximately 1000 MPa according to the mechanical test.

### 2.3 Surgical technique and follow-up

**FIGURE 5:**
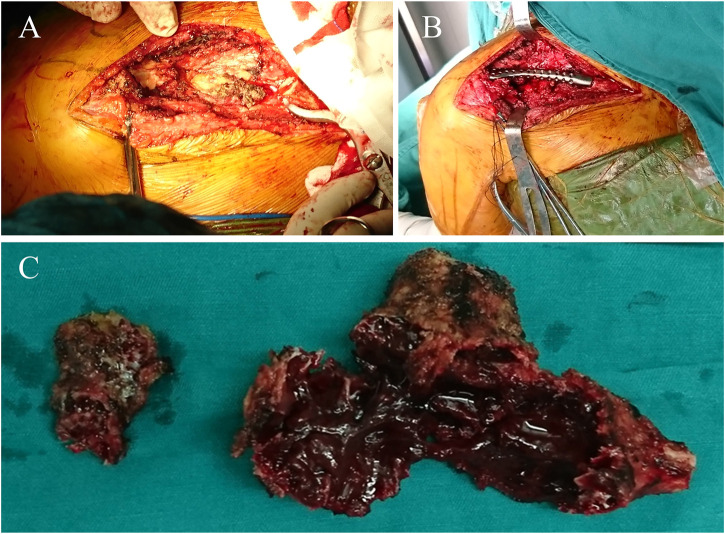
Intraoperative pictures. **(A)** Resection of the lateral clavicle was performed. **(B)** Implantation of the prosthesis. **(C)** Photograph of the resected tumor specimens.

The surgery was performed by the senior surgeon (CQ T). After general anesthesia, the patient was placed in a supine position and the right shoulder was elevated with padding. A curvilinear extensile approach following the bone outline about 15 cm long was utilized. The tissue was carefully disinfected, and the clavicle was sufficiently exposed. The deltoideus and trapezius muscles were cut from the lateral clavicle. And then, the acromioclavicular ligament and coracoclavicular ligament were cut off. The clavicle tumor resection was performed precisely according to the preoperative plan (Figure 5). Then, the coracoid process was resected. The next step was the implantation of the prosthesis. The residual medial clavicle was reamed, and the curved intramedullary stem was inserted. The flat fin was inserted under acromion. After implantation of the prosthesis, the soft tissue was repaired by suture holes of the prosthesis. The operating time was 2h, the blood loss was 200mL, and there were no intraoperative complications.

**FIGURE 6:**
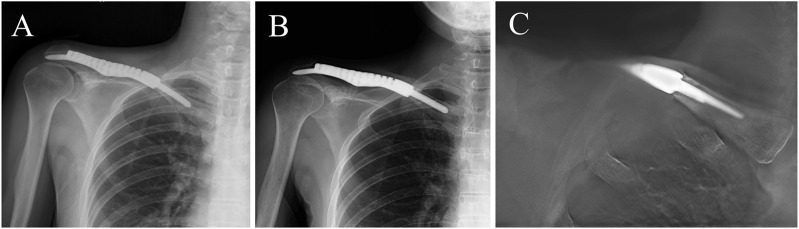
**(A**, **B)** 12-month and 36-month postoperative anteroposterior view X-ray. **(C)** The anteroposterior digital tomosynthesis graph showed no radiolucent lines around the intramedullary stem.

After the surgery, the patient was asked to wear an arm sling for 2 weeks. Thereafter, the passive exercise of the shoulder was allowed. Active motion was started at 3 weeks postoperatively. The strengthening and resistive exercises of the shoulder girdle commenced at 6 weeks postoperatively and progressed gradually depend on the tolerance of patient. Postoperatively radiographic examinations revealed a good position of the prosthesis, neither breakage nor loosening was detected (Figure 6). The right shoulder mobility returned to approximate level of preoperative shoulder 2 months after surgical reconstruction, with the range of motion (ROM) of flexion 80°, extension 40°, abduction 80°, adduction 30°, external rotation 55°, and internal rotation 60°. The patient maintained the normal shoulder function during the 48 months follow-up period. There was no pain during shoulder motion. The Musculoskeletal Tumor Society Score (MSTS) score was 29 and the Functional Evaluation Form recommended by the American Shoulder and Elbow Surgeons (ASES) score was 95.

## 3 Discussion and conclusions

Intraosseous hemangiomas are uncommon benign bone tumors. In this case, lateral clavicle and coracoid process were involved meanwhile, which is very rare. The patient underwent surgical resection of the lesions and reconstruction of the clavicle bone defect with a 3D-printed personalized prosthesis. This procedure subsequently yielded satisfactory outcomes in terms of cosmetic and postoperative shoulder function.

Primary bone tumors involving the clavicle are rare (Korkmaz et al., 2022), and partial or total claviculectomy is considered the preferred treatment option (Liu et al., 2019). However, whether or not the clavicle should be reconstructed is still controversial. Rossi et al. and Li et al. observed only mild functional deficit and few postoperative complications in patients who underwent partial or total claviculectomy without reconstruction (Li et al., 2011; Rossi et al., 2011). In contrast, Rubright et al. concluded that patients without reconstruction gradually lost compensatory ability with time after claviculectomy, and complained diminished strength of shoulder motion and scapular dyskinesis for long-term follow-up (Rubright et al., 2014). Similar results were reported by Lin et al. and Liu et al., claviculectomy without reconstruction resulted in many complications, such as sloped shoulder, postoperative pain, limited motion of the shoulder joint, and restriction of daily activities (Lin et al., 2014; Liu et al., 2019). Additionally, the risk of subclavicle vascular injury and brachial plexus damage would increase because of the diminishing of protective effects after claviculectomy. Therefore, reconstruction of the bone defect after claviculectomy is suggested to maintain the normal shoulder function and reduce the incidence of complications. In the present case, the patient underwent reconstruction with 3D-printed personalized prosthesis after tumor resection of the lateral clavicle. During the 48 months follow-up, the patient maintained normal shoulder function, and no adverse effects on daily activities were observed.

Besides reconstruction of the clavicle bone defect, restoration of the shoulder support structure is necessary to maintain the normal shoulder joint function for long-term. Momberger et al. reported success restoration of the shoulder support by allograft in two patients; unfortunately the allografts were taken out due to severe shoulder pain and non-union (Momberger et al., 2000). On the contrary, no signs of complication were detected in the present case after implantation of the 3D-printed personalized prosthesis. And the patient maintained normal shoulder joint function. In addition, bone cement modeling was another alternative method to restore the shoulder support with the advantages of simple procedure (Vartanian et al., 2006; Lin et al., 2014). However, bone cement modeling cannot achieve osseointegration because of lack bioactivity, and anatomical match between the implant and host hone is poor. In the present case, the prosthesis designed by mirroring the normal clavicle was close to the personal anatomy. Meanwhile, in our case, after the subtotal clavicle resection, fixation of the bone cement modeling might be difficult, and postoperative complications such as dislocation might occur. To our best knowledge, there were three studies regarding the use of 3D-printed prosthesis to reconstruct clavicle (Fan et al., 2015; Abdalbary et al., 2021; Chen et al., 2021), and among them only one study reported reconstruction of the lateral clavicle similar to our patient (Abdalbary et al., 2021). However, the implant was a tubular prosthesis, covering the medial clavicle stump. Despite this tubular prosthesis restored the shoulder support, the mismatch size between the prosthesis and residual clavicle cannot be avoided. In addition, the larger size of prosthesis affected the symmetry of body both sides, and thus impaired the cosmetic. In the present case, 3D-printed personalized prosthesis with anatomy-confirmed shape restored the normal clavicle counter, without impairment on cosmetic. Furthermore, our prosthesis was designed with a flat fin inserted under the acromion, increasing support effect for shoulder joint.

Aseptic loosening is a common reason leading to failure of prosthetic reconstruction. In the present case, a curved intramedullary stem was inserted into medial clavicle stum. Although the stem was truly short, the prosthesis was well in position and no loosening was observed during the 48 months follow-up. The curved stem was compatible with clavicle anatomy shape, allowing for better coordination to limb biomechanics.

There are also certain shortcomings of this surgical technique. Firstly, the prosthesis is custom made and therefore more expensive. Secondly, the prosthesis was designed based on patient’s CT scans, and if the patient has any contraindications for CT scans, this technique will be limited.

In conclusion, the application of 3D-printed personalized prosthesis reconstructed the supporting structure of the shoulder. And then, the normal function of the shoulder joint is maintained and adverse effects on daily activities are avoided after claviculectomy. 3D-printed personalized prosthesis seems to be a good option to reconstruct the lateral clavicle bone defect after tumor resection.

## Data Availability

The raw data supporting the conclusions of this article will be made available by the authors, without undue reservation.

## Abbreviations

3D, Three-dimensional; CT, Computed tomography; MRI, Magnetic resonance imaging; MSTS, Musculoskeletal Tumor Society; ASES, American Shoulder and Elbow Surgeons; ROM, Range of motion.
